# *Brucella abortus* induces TNF-α-dependent astroglial MMP-9 secretion through mitogen-activated protein kinases

**DOI:** 10.1186/1742-2094-10-47

**Published:** 2013-04-12

**Authors:** M Cruz Miraglia, Romina Scian, Clara García Samartino, Paula Barrionuevo, Ana M Rodriguez, Andrés E Ibañez, Lorena M Coria, Lis N Velásquez, Pablo C Baldi, Juliana Cassataro, M Victoria Delpino, Guillermo H Giambartolomei

**Affiliations:** 1 Laboratorio de Inmunogenética, Instituto de Inmunología, Genética y Metabolismo, Hospital de Clínicas “José de San Martín”, (CONICET/UBA), Buenos Aires, Argentina; 2 Instituto de Estudios de la Inmunidad Humoral (CONICET/UBA), Facultad de Farmacia y Bioquímica, Universidad de Buenos Aires (UBA), Buenos Aires, Argentina

**Keywords:** *Brucella abortus*, Neurobrucellosis, Astrocytes, Matrix metalloproteinases, MAPK, TNF-α, Lipoproteins

## Abstract

**Background:**

Central nervous system (CNS) invasion by bacteria of the genus *Brucella* results in an inflammatory disorder called neurobrucellosis. We have recently demonstrated that *B. abortus* infects microglia and astrocytes, eliciting the production of a variety of pro-inflammatory cytokines which contribute to CNS damage. Matrix metalloproteinases (MMP) have been implicated in inflammatory tissue destruction in a range of pathological situations in the CNS. Increased MMP secretion is induced by pro-inflammatory cytokines in a variety of CNS diseases characterized by tissue-destructive pathology.

**Methods:**

In this study, the molecular mechanisms that regulate MMP secretion from *Brucella*-infected astrocytes *in vitro* were investigated. MMP-9 was evaluated in culture supernatants by ELISA, zymography and gelatinolytic activity. Involvement of mitogen-activated protein kinases (MAPK) signaling pathways was evaluated by Western blot and using specific inhibitors. The role of TNF-α was evaluated by ELISA and by assays with neutralizing antibodies.

**Results:**

*B. abortus* infection induced the secretion of MMP-9 from murine astrocytes in a dose-dependent fashion. The phenomenon was independent of bacterial viability and was recapitulated by L-Omp19, a *B. abortus* lipoprotein model, but not its LPS. *B. abortus* and L-Omp19 readily activated p38 and Erk1/2 MAPK, thus enlisting these pathways among the kinase pathways that the bacteria may address as they invade astrocytes. Inhibition of p38 or Erk1/2 significantly diminished MMP-9 secretion, and totally abrogated production of this MMP when both MAPK pathways were inhibited simultaneously. A concomitant abrogation of *B. abortus*- and L-Omp19-induced TNF-α production was observed when p38 and Erk1/2 pathways were inhibited, indicating that TNF-α could be implicated in MMP-9 secretion. MMP-9 secretion induced by *B. abortus* or L-Omp19 was completely abrogated when experiments were conducted in the presence of a TNF-α neutralizing antibody. MMP-9 activity was detected in cerebrospinal fluid (CSF) samples from patients suffering from neurobrucellosis.

**Conclusions:**

Our results indicate that the inflammatory response elicited by *B. abortus* in astrocytes would lead to the production of MMP-9 and that MAPK may play a role in this phenomenon. MAPK inhibition may thus be considered as a strategy to control inflammation and CNS damage in neurobrucellosis.

## Background

Human brucellosis is a protean disease with a diversity of clinical signs and symptoms resulting from infection with *Brucella* species [1]. It is chiefly an inflammatory disease. Inflammation is present both in the acute and chronic phases of the disease and in virtually all of the organs affected. Clinical signs of such inflammation are undulant fever, endocarditis, arthritis, osteomyelitis, meningitis, pleocytosis, cellular infiltration of the joints, orchitis, nephritis, hepatic granuloma, etc [2]. In the central nervous system (CNS), where the function of neurons is normally protected by the maintenance of an antiinflammatory environment [3], infection with *Brucella* leads to an inflammatory disorder called neurobrucellosis which involves tissue destruction [4,5]. The underlying mechanisms of this phenomenon are currently unclear. Yet, a better understanding of the pathogenesis of neurobrucellosis would help if improvements are to be made on therapies that help to cure or ameliorate this form of the disease [6].

Matrix metalloproteinases (MMP) are a family of calcium- and zinc-dependent proteinases that have been implicated in inflammatory tissue destruction in a range of pathological situations in the CNS, including experimental autoimmune encephalomyelitis, multiple sclerosis, and CNS tuberculosis [7-10]. Particularly, MMP-9 can degrade many structural components of the blood-brain barrier and CNS tissue matrix, including type IV collagen, laminins, and fibronectin [11,12]. MMP-9 can also mediate direct damage to neurons [13] and MMP-9 knockout mice are protected against ischemic and post-traumatic damage which follows blood-brain barrier disruption [14]. In addition, MMP have been implicated in tissue-destructive pathology in osteoarticular brucellosis [15-18].

Astrocytes are the most numerous cell type within the CNS, outnumbering neurons by a factor of ten. They are integral to both maintenance of the CNS tissue matrix and innate immunity within the CNS [19], and also the well-being of the blood-brain barrier [20,21]. In normal physiology MMP-9 secretion is highly regulated, and under these conditions astrocyte-derived MMP-9 participates in tissue remodeling and neurite extension [22,23]. Yet, astrocyte-derived MMP-9 may contribute to the development of a tissue-destructive phenotype in the CNS. Increased MMP-9 secretion is induced by pro-inflammatory cytokines in a range of CNS diseases characterized by tissue-destructive pathology [24].

We have recently demonstrated that upon infection with *B. abortus*, astrocytes have a key role in eliciting a pro-inflammatory cytokine response (TNF-α, IL-1β and IL-6) that leads to astrogliosis *in vivo* and *in vitro*[25]. However, the role that astrocytes play in control of MMP-9 secretion and its potential in inflammatory tissue destruction in neurobrucellosis is unknown.

Mitogen-activated protein kinases (MAPK) play a key role in the regulation of neuronal development, growth, and survival, as well as in pro-inflammatory cytokine production. The Erk1/2 pathway is mainly associated with neuronal development, whereas the p38 kinase is a stress-activated kinase that participates in the regulation of pro-inflammatory cytokines and apoptosis [26]. Erk1/2 have been implicated in astrogliosis [27,28], and bacterial lipopolysaccharide (LPS) has been shown to induce IL-6 and TNF-α in both astrocytes and microglia by activating Erk1/2 and p38 [29]. Moreover, *Mycobacterium tuberculosis* regulates MAPK-dependent astrocyte MMP-9 secretion [30].

In this study, we investigated the cytokine network that regulates MMP secretion from *Brucella*-infected astrocytes and examined the MAPK signaling pathways involved. Results are discussed in the context of CNS damage in neurobrucellosis.

## Methods

### Animals

For the primary cultures of astrocytes, one- to three-day-old BALB/c mice (Instituto de Estudios de la Inmunidad Humoral (IDEHU), Facultad de Farmacia y Bioquímica, Universidad de Buenos Aires, Buenos Aires, Argentina) were used. Animals were obtained from breeding couples housed under controlled temperature (22°C ± 2°C) and artificial light under a 12-hour cycle period. Mice were kept under specific pathogen-free conditions in a positive-pressure cabinet (EHRET, Emmendingen, Germany) and provided with sterile food and water *ad libitum*. All animal procedures were performed according to the rules and standards for the use of laboratory animals of the National Institute of Health, USA. Animal experiments were approved by the ethics committee of the IDEHU Institute.

### Bacterial culture

*B. abortus* S2308, *B. canis*, *B. melitensis* H38 and *B. suis* 1330 were grown overnight in 10 ml of tryptic soy broth (TSB) with constant agitation at 37°C. Bacteria were harvested by centrifugation for 15 minutes at 6,000 × *g* at 4°C and washed twice in 10 ml of phosphate-buffered saline (PBS). Bacterial numbers in the cultures were estimated by comparing the optical densities at 600 nm with a standard curve obtained in our laboratory. To prepare inocula, cultures were diluted in sterile PBS to the desired bacterial concentration on the basis of the optical density readings, but the precise concentrations of inocula were determined by plating cells onto tryptic soy agar. To obtain heat-killed *B. abortus* (HKBA), bacteria were washed five times for 10 minutes each in sterile PBS, heat-killed at 70°C for 20 minutes, aliquoted, and stored at −70°C until they were used. The total absence of *B. abortus* viability after heat killing was verified by the absence of bacterial growth on tryptic soy agar. All live *Brucella* manipulations were performed in biosafety level 3 facilities.

### Lipoproteins and LPS

*B. abortus* lipidated outer membrane protein 19 (L-Omp19) and unlipidated Omp19 (U-Omp19) were obtained as described [31]. Both recombinant proteins contained less than 0.25 endotoxin U/μg of protein as assessed by *Limulus* Amebocyte Lysates (Associates of Cape Cod Inc., MA, USA). *B. abortus* S2308 LPS was provided by I. Moriyon. The synthetic lipohexapeptide (tripalmitoyl-S-glyceryl-Cys-Ser-Lys4-OH (Pam_3_Cys)) was purchased from Boehringer Mannheim (Mannheim, Germany).

### Primary astrocyte culture

Highly pure astrocytes (> 95%) were established from primary mixed glial cultures obtained from the forebrain of one- to three-day-old BALB/c mice following previously published procedures [25].

### *In vitro* infection

Astrocytes were cultured in 24 well plates at a density of 5 × 10^5^ cells per well in DMEM high glucose (Hyclone, Logan, Utah, USA) containing 10% heat inactivated FBS (GIBCO BRL, Life Technologies, Grand Island, NY, USA), supplemented with 2 mM L-glutamine and 1 mM sodium without the addition of antibiotics. Cells were infected with *B. abortus* (at different multiplicities of infection (MOI)), *B. melitensis*, *B. canis* or *B. suis* (MOI 100: 1) for two hours in medium containing no antibiotics. Astrocytes were extensively washed to remove un-internalized bacteria, and infection was maintained for different times in the presence of 100 μg/ml gentamicin and 50 μg/ml streptomycin to kill remaining extracellular bacteria. Cells were washed three times with PBS before processing. To monitor *Brucella* intracellular survival, infected cells were lysed with 0.1% (v/v) Triton X-100 in H_2_O after PBS washing and serial dilutions of lysates were plated onto TSB agar plates to enumerate colony forming units (CFUs).

### Assessment of cytokines and MMP secretion

Primary astroglial-enriched cultures were either infected with different MOI or stimulated for 48 hours with *B. abortus* LPS (1,000 ng/ml), HKBA (1 × 10^6^ to 1 × 10^9^ bacteria/ml), U-Omp19 (1,000 ng/ml), L-Omp19 (10 to 1,000 ng/ml), Pam_3_Cys (25 ng/ml), TNF-α (5 ng/ml) (Pharmingen, San Diego, CA, USA) or phorbol myristate acetate (PMA) (50 ng/ml). Secretion of TNF-α in the supernatants was quantified by ELISA (Pharmingen, San Diego, CA, USA). MMP was determined by ELISA, zymography and gelatinolytic activity.

### Zymography

Gelatinase activity was assayed by the method of Hibbs *et al*. [32], which was standardized by others [33-35]. Briefly, a total of 20 μl of cell culture supernatants from infected astrocytes or from untreated controls, as well as cerebrospinal fluid from patients with neurobrucellosis were mixed with 5 μl of 5X loading buffer (0.25 M Tris (pH 6.8), 50% glycerol, 5% SDS, and bromophenol blue crystals) and loaded onto 10% SDS-PAGE gels containing 1 mg/ml gelatin (Sigma-Aldrich, Buenos Aires, Argentina). Following electrophoresis, gels were washed with a solution containing 50 mM Tris-HCl (pH 7.5) and 2.5% Triton X-100 (buffer A) for 30 minutes and with buffer A added with 5 mM CaCl_2_ and 1 μM ZnCl_2_ for 30 minutes and were later incubated with buffer A with additional 10 mM CaCl_2_ and 200 mM NaCl for 48 hours at 37°C. This denaturation/renaturation step promotes MMP activity without the proteolytic cleavage of pro-MMP. Gelatin activity was visualized by the staining of the gels with 0.5% Coomassie blue. Unstained bands indicated the presence of gelatinase activity, and their positions indicated the molecular weights of the enzymes involved. The identity of the candidate MMP was confirmed by a specific ELISA.

### Measurement of MMP-9 levels

MMP-9 levels present in culture supernatants from astrocytes were quantified by sandwich ELISA using paired MMP-9-specific monoclonal antibodies according to the manufacturer’s instructions (R&D Systems, Minneapolis, MN, USA).

### Gelatinase activity under native conditions

Gelatinase activity in unprocessed culture supernatants (native conditions) was measured by using a gelatinase/collagenase fluorometric assay kit (EnzChek; Invitrogen, Carlsbad, CA, USA) according to the manufacturer’s instructions. The EnzChek kit contains DQ gelatin, a fluorescein-conjugated gelatin so heavily labeled with fluorescein that fluorescence is quenched. When this substrate is digested by gelatinases or collagenases, it yields highly fluorescent peptides, and the fluorescence increase is proportional to the proteolytic activity. Collagenase purified from *Clostridium histolyticum* provided in the assay kit served as a control enzyme. Plates were read with a fluorescence plate reader (Victor3; Perkin-Elmer, Waltham, MA, USA).

### Signaling pathways

To study the potential involvement of different signaling pathways in the production of MMP-9 and TNF-α by astrocytes, pharmacological inhibitors (SB203580, a p38 MAPK inhibitor; PD98059, an Erk1/2 MAPK inhibitor; and SP600125, a Jnk1/2 inhibitor) or vehicle (dimethyl sulfoxide (DMSO)) were added two hours before the beginning of infection or stimulation and kept throughout. SB203580, PD98059 and SP600125 (Calbiochem, San Diego, CA, USA) were used at a concentration of 6 μM, 50 μM and 10 μM, respectively; based on previous reports [36]. Cell viability after incubation with these inhibitors was higher than 90%, as assessed by staining with trypan blue. To account for any possible effect of DMSO on cell viability, cell cultures not treated with the inhibitors were treated with the highest final concentration of DMSO used in these studies (0.01%), and the results were compared with those of cell cultures not exposed to DMSO.

### p38, Erk1/2 and Jnk1/2 activation by Western blot

Astrocytes treated with HKBA or L-Omp19 or stimulated with PMA (positive control) were lysed in ice-cold lysis buffer consisting of 1% Triton X-100 in 150 mM NaCl, 25 mM Tris-HCl (TBS) pH 7.4, and protease and phosphatase inhibitor cocktails (Sigma-Aldrich, Buenos Aires, Argentina). Lysates were incubated on ice for 10 minutes and cleared by centrifugation at 13,000 × g for 10 minutes. Protein concentration was determined by the bicinchoninic acid method (Pierce, Rockford, IL, USA) using bovine serum albumin as standard, and equal amounts of proteins were loaded onto electrophoresis gels. After separation, proteins were transferred to a nitrocellulose membrane (GE Healthcare, Little Chalfont, UK) and blocked for one hour with 0.1% Tween-20. Then, membranes were incubated with primary anti-Erk1/2 and anti-p38 antibodies (total and phosphorylated) (Santa Cruz Biotechnology, Santa Cruz, CA, USA) (1:1,000 dilution) or anti-Jnk1/2 antibodies (Cell signaling Technology, Danvers, MA, USA) (1:1,000 dilution) overnight at 4°C. After washing, the membrane was incubated with a 1:2,000 dilution of peroxidase-conjugated secondary antibody (Santa Cruz Biotechnology, Santa Cruz, CA, USA) for one hour. Protein bands were visualized on ECL® (GE Healthcare, Little Chalfont, UK) Hyperfilm by chemiluminescence.

### Blocking of TNF-α

Neutralization experiments were performed with 20 μg/ml of anti-TNF-α neutralizing antibody (clone MP6-XT3) or its isotype control (Pharmingen, San Diego, CA, USA).

### CSF and serum samples

CSF and serum samples were obtained from a) non-infected controls (patients suspected of suffering Alzheimer’s disease), b) patients who had neurobrucellosis (as shown by signs and symptoms indicative of neurological involvement, the isolation of *Brucella* spp. from CSF samples, and the detection of anti-*Brucella* antibodies in CSF by an agglutination or Coombs test), c) a patient who had brucellosis without neurological involvement (this patient had signs and symptoms typical of brucellosis and was positive by standard *Brucella* serology, and had severe headaches that motivated the extraction of a CSF sample to rule out neurobrucellosis; however, this CSF sample had normal physicochemical parameters, no microorganism was isolated and no anti-*Brucella* antibodies were detected by agglutination); d) CSF samples from patients who had meningitis caused by infectious agents other than *Brucella* spp. (*Staphylococcus aureus* and *Streptococcus pneumoniae*). A written consent was obtained from all patients and all procedures were approved by the ethics committee of the IDEHU Institute. Antibodies against *Brucella* cytoplasmic proteins and LPS were detected in CSF and serum samples as described [37]. MMP-9 in CSF samples was detected by zymography.

### Statistical analysis

Statistical analysis was performed with one-way ANOVA, followed by *post hoc* Tukey test using GraphPad Prism 4.0 software, San Diego, CA, USA). Data was represented as mean ± standard error of the mean (SEM).

## Results

### *B. abortus* infection induces MMP-9 secretion from astrocytes

**Figure 1:**
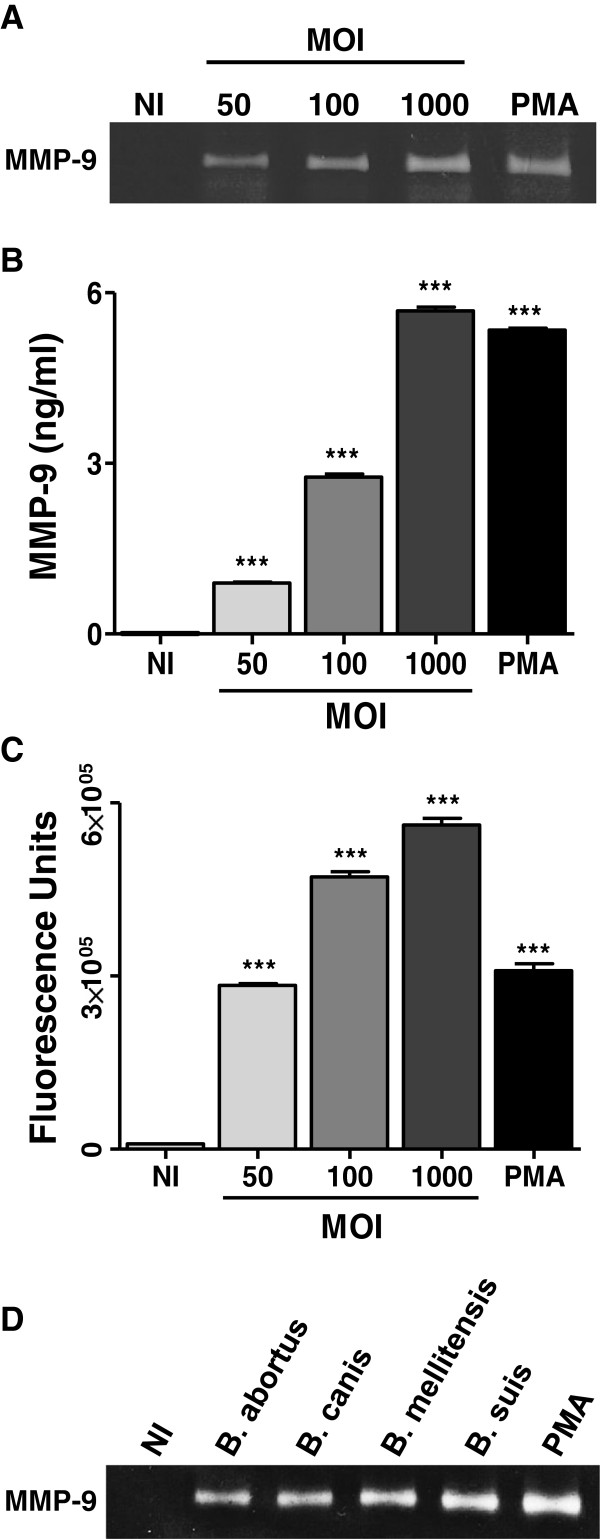
***B. abortus *induces secretion of MMP-9 from astrocytes.** Astrocytes were infected with *B. abortus* at different multiplicities of infection (MOI). MMP-9 production was determined by zymography (**A**) and ELISA (**B**) at 48 hours post infection. For MMP activities, the supernatants from infected and non-infected astrocytes were incubated with a fluorescein-conjugated gelatin substrate that produces highly fluorescent peptides when gelatin is digested. Data are expressed in fluorescence units informed by the fluorometer (**C**). MMP-9, as determined by zymography, was also induced in astrocytes infected with *B. canis*, *B. melitensis* and *B. suis* (**D**). Phorbol myristate acetate (PMA) was used as a positive control. Bars express the mean ± SEM of duplicates. Data shown are from a representative experiment of three performed. ***P < 0.001 versus non-infected (NI) astrocytes.

We have previously demonstrated the ability of *B. abortus* to invade and replicate in primary cultures of mouse astrocytes [25]. As mentioned, mouse astrocytes can respond to bacterial infections with an enhanced secretion of MMP [30]. Thus, we decided to investigate whether *B. abortus* infection induces MMP expression in mouse astrocytes. Astrocyte infection resulted in an increased MMP activity that was detected by zymography in the supernatants of infected cells at 48 hours post infection, which according to the molecular weight of the band corresponded to MMP-9 (Figure 1A). This was confirmed by ELISA, which revealed significantly (*P* < 0.001) increased levels of MMP-9 in supernatants of infected cells as compared to uninfected cells (Figure 1B). *In vivo*, the activity of MMP is counterbalanced by the activity of tissue inhibitors including tissue inhibitors of metalloproteinases (TIMP) [38]. Therefore, the net gelatinase or collagenase activity in a complex sample, such as culture supernatants, depends on the balance between MMP and TIMP activities. This net activity is not revealed by zymographic methods, since MMP-TIMP complexes may dissociate during gel electrophoresis. To assess whether an increased net gelatinase activity is generated by *Brucella*-infected astrocytes, culture supernatants from these cells were incubated with a non-fluorescent gelatin-fluorescein conjugate, and the fluorescence unmasked as a consequence of gelatin degradation was measured in a fluorometer. The enzymatic activity (measured as fluorescence intensity) increased significantly (*P* < 0.001) in supernatants of *Brucella*-infected astrocytes as compared to uninfected cells (Figure 1C). By all methods employed, the magnitude of MMP-9 released to culture supernatants was directly related to the multiplicities of infection (MOI) used to infect cells. MMP-9 secretion was not a unique attribute of *B. abortus* since astrocytes infected with other *Brucella* species such as *B. canis*, *B. melitensis* and *B. suis*, were able to induce MMP-9 secretion (Figure 1D). These results indicate that *Brucella* infection induces the secretion of MMP-9 in mouse astrocytes.

### *B. abortus*-induced secretion of MMP-9 in astrocytes is mediated by L-Omp19, but not by *B. abortus* LPS

**Figure 2:**
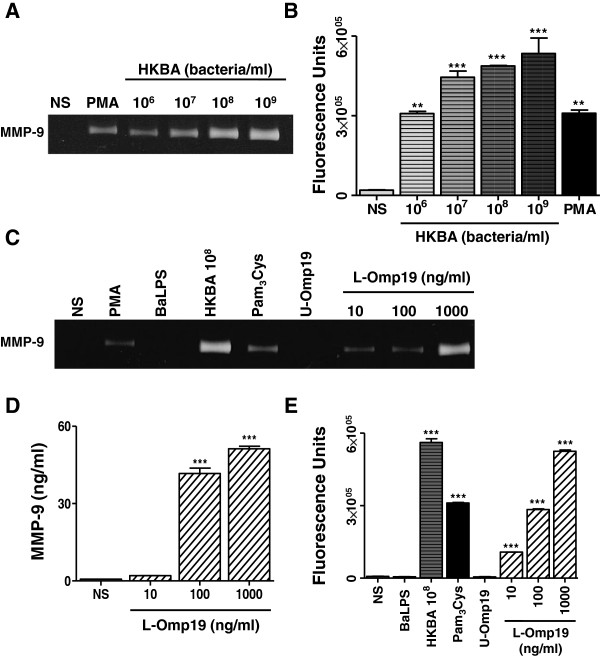
**HKBA and L-Omp19 induce MMP-9 secretion from astrocytes.** Astrocytes were stimulated with HKBA (from 10^6^ to 10^9^ bacteria/ml), *B. abortus* (Ba) LPS (Ba , 1,000 ng/ml), lipidated Omp19 (L-Omp19, from 10 to 1,000 ng/ml) or unlipidated Omp19 (U-Omp19, 1,000 ng/ml). MMP-9 production was determined by zymography (**A**, **C**) and ELISA (**D**) at 48 hours post stimulation. For MMP activities, culture supernatants were incubated with a fluorescein-conjugated gelatin substrate that produces highly fluorescent peptides when gelatin is digested (**B**, **E**). PMA (50 ng/ml) and Pam_3_Cys (50 ng/ml) were used as positive controls. Bars express the mean ± SEM of duplicates. Data shown are from a representative experiment of four performed. ***P < 0.001; **P < 0.01 versus non-stimulated (NS) astrocytes.

To test whether viable bacteria were necessary to induce MMP from astrocytes, the ability of heat-killed *B. abortus* (HKBA) to induce the secretion of MMP-9 was examined. PMA was used as control. The secretion of MMP-9 was markedly enhanced in culture supernatants from astrocytes stimulated with HKBA when compared with the unstimulated cells, as assessed by zymography and gelatinolytic activity test (Figure 2A and B, respectively). MMP-9 production was a function of the amount of bacteria present in the culture. A significant (*P* < 0.01) MMP-9 activity was detected in cultures containing between 1 × 10^6^ and 1 × 10^9^ bacteria/ml, a similar bacterial concentration that the one able to elicit the secretion of MMP-9 with live bacteria. These results suggest that the secretion of MMP-9 could be induced by a structural component of *B. abortus*. Since we have previously demonstrated that *B. abortus* lipoproteins induce cytokine and MMP secretion in different cells types [15,17,18,25], we hypothesized that lipoproteins could also mediate such effects in astrocytes. To investigate this hypothesis we used recombinant L-Omp19 as a *Brucella* lipoprotein model [31]. Astrocytes were incubated with L-Omp19 and culture supernatants were harvested 48 hours later to measure the secretion of MMP-9 by zymography, ELISA and gelatinolytic activity test. L-Omp19 induced a significant (*P* < 0.001) secretion of MMP-9 in a dose-dependent fashion (Figure 2C-E). Independently of the way in which MMP-9 activity was evaluated, its induction was dependent on the lipidation of L-Omp19, as U-Omp19 failed to induce the secretion of MMP-9 (Figure 2C and E). To ascertain whether the effects elicited by L-Omp19 could be extended to all *B. abortus* lipoproteins, the production of MMP-9 was also evaluated in astrocytes incubated with a synthetic lipohexapeptide (Pam_3_Cys) that mimics the structure of the lipoprotein lipid moiety. Pam_3_Cys also stimulated MMP-9 secretion by astrocytes (Figure 2C and E). These results indicate that the Pam_3_-modified cysteine is the molecular structure of L-Omp19 that induces MMP-9 secretion. At variance with the results obtained with L-Omp19, *B. abortus* LPS did not induce MMP-9 production even when used at high doses (1,000 ng/ml) (Figure 2C and E). Altogether, these results indicate that *B. abortus* lipoproteins induce the secretion of MMP-9 in astrocytes.

### HKBA and L-Omp19 induce phosphorylation of p38 and Erk1/2; but not Jnk1/2 in astrocytes

**Figure 3:**
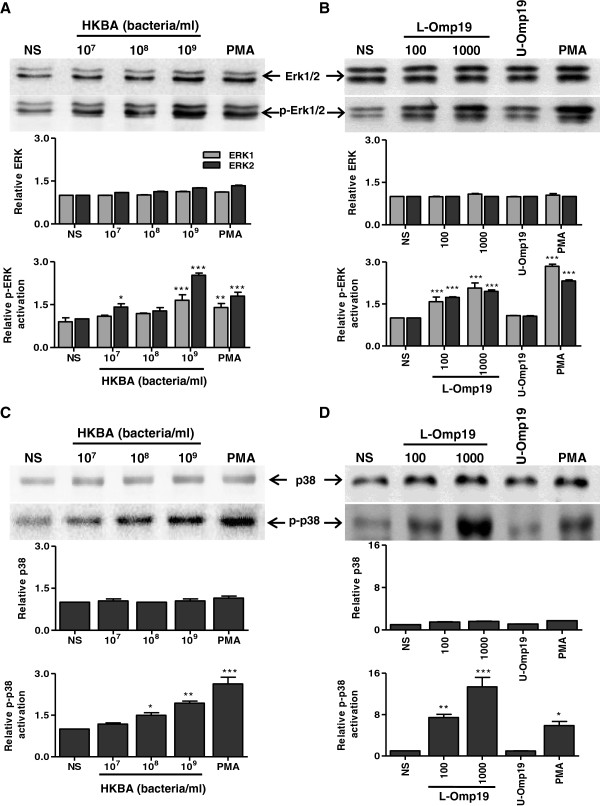
**HKBA and L-Omp19 induce p38 and Erk1/2 phosphorylation in astrocytes.** Astrocytes were stimulated with HKBA (**A**, **C**), L-Omp19 or U-Omp19 (1,000 ng/ml) (**B**, **D**). MAPK phosphorylation was determined by Western blot. Total and phosphorylated Erk1/2 (**A**, **B**, upper panels). Total and phosphorylated p38 (**C**, **D**, upper panels). PMA was used as a positive control. Densitometric analysis of results from three independent experiments as performed in (A-D), lower panels. *P < 0.1, **P < 0.01, ***P < 0.001 for comparisons with non-stimulated (NS) astrocytes. The data shown are from a representative experiment of three performed.

**Figure 4:**
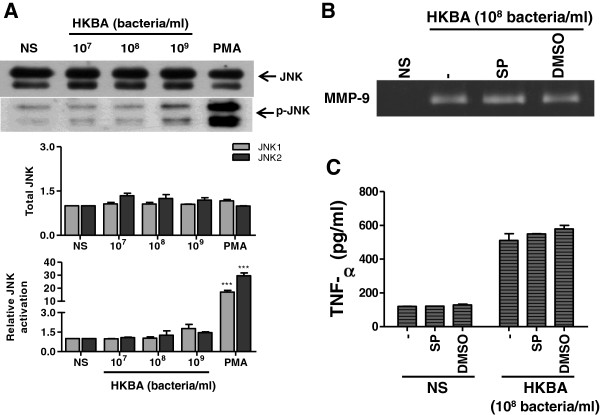
**HKBA does not activate Jnk1/2.** Astrocytes were stimulated with HKBA. Jnk1/2 phosphorylation was determined by Western blot. Total and phosphorylated Jnk1/2 (**A**, upper panel). PMA was used as a positive control. Densitometric analysis of results from three independent experiments as performed in (A, lower panel). Astrocytes were incubated with Jnk1/2 inhibitor (SP) two hours before the beginning of culture and kept throughout. Culture supernatants were harvested at 48 hours post stimulation with HKBA to assess the expression of MMP-9 by zymography (**B**) or TNF-α by ELISA (**C**). Bars express the mean ± SEM of duplicates. Data shown are from a representative experiment of three performed. NS: non-stimulated astrocytes. ***P < 0.001 versus non-stimulated (NS) astrocytes.

MAPK play a key role in the regulation of pro-inflammatory cytokines and MMP production [30,39]. Thus, we explored the possibility that MAPK could play a role in mediating MMP-9 secretion, as induced by *B. abortus* lipoproteins. As a first step, we investigated whether p38 and Erk1/2 MAPK were phosphorylated in astrocytes when these cells were treated with HKBA or L-Omp19. PMA stimulation was used as a positive control. Both, HKBA and L-Omp19 induced p38 and Erk1/2 phosphorylation in a dose-dependent fashion (Figure 3). L-Omp19-induced phosphorylation depended upon the lipidation of L-Omp19, as U-Omp19 failed to induce the activation of both p38 and Erk1/2 MAPK. On the contrary, HKBA induced an increase in Jnk1/2 phosphorylation only at the highest concentration of HKBA tested, albeit not significantly (Figure 4A).

### p38 and Erk1/2 signaling pathways are involved in the secretion of MMP-9 and TNF-α induced by HKBA and L-Omp19 in astrocytes

**Figure 5:**
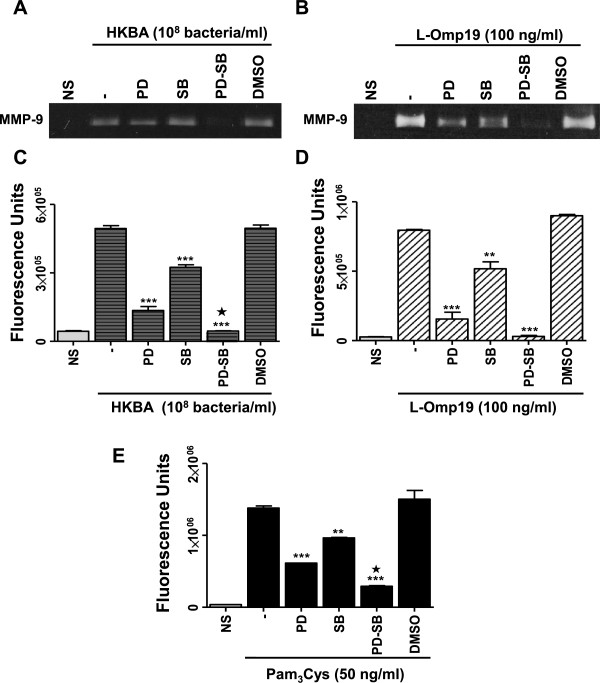
**Erk1/2 and p38 MAPK pathways are involved in MMP-9 secretion by HKBA- or L-Omp19-stimulated astrocytes.** Astrocytes were incubated with inhibitors of MAPK pathways (PD: inhibitor of Erk1/2; SB: inhibitor of p38) two hours before the beginning of culture and kept throughout. Culture supernatants were harvested at 48 hours post stimulation with HKBA, L-Omp19 and Pam_3_Cys to assess the expression of MMP-9 by zymography (**A**) and (**B**). The net gelatinase activity was expressed in fluorescence units informed by the fluorometer (**C**, **D** and **E**). Pam_3_Cys was used as a positive control. Bars express the mean ± SEM of duplicates. Data shown are from a representative experiment of four performed. NS: non-stimulated astrocytes. *P < 0.05, **P < 0.01, ***P < 0.001 versus untreated (minus sign); and *P* < 0.05 versus the treatment with PD or SB alone (black star).

**Figure 6:**
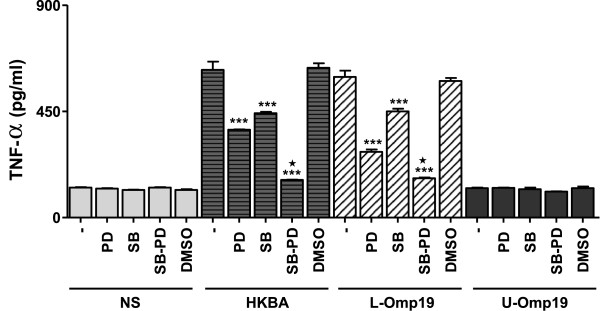
**Erk1/2 and p38 MAPK pathways are involved in TNF-α secretion by HKBA- or L-Omp19-stimulated astrocytes.** Astrocytes were incubated with inhibitors of MAPK pathways (PD: inhibitor of Erk1/2; SB: inhibitor of p38) two hours before the beginning and kept throughout the culture with HKBA (10^8^ bacteria/ml), L-Omp19 (1,000 ng/ml) and U-Omp19 (1,000 ng/ml). Culture supernatants were harvested at 48 hours post stimulation to assess the secretion of TNF-α by ELISA. Bars express the mean ± SEM of duplicates. Data shown are from a representative experiment of four performed. NS: non-stimulated astrocytes. ***P < 0.001 versus untreated (minus sign); *P* < 0.001 versus the treatment with PD or SB alone (black star).

We next investigated whether the specific inhibition of p38 and Erk1/2 MAPK could inhibit MMP-9 production. Therefore, inhibition experiments of the p38 and Erk1/2 MAPK signaling pathways were performed with the specific inhibitors SB203580 and PD98059, respectively. Both, p38 and Erk1/2 MAPK pathways participated in the production of MMP-9 as elicited by HKBA and L-Omp19. By the zymography or gelatinolytic activity test, the production of MMP-9 was significantly inhibited (*P* < 0.001) either by p38 or Erk1/2 inhibitors, and was completely abrogated when both inhibitors were used together (Figure 5A-D). This inhibitory effect was reproduced when astrocytes were stimulated with Pam_3_Cys (Figure 5E). Conversely, inhibition of Jnk1/2 with the specific inhibitor SP600125 had no effect on HKBA-induced MMP-9 production (Figure 4B). We had previously established that *Brucella* lipoproteins induced TNF-α production by astrocytes [25]. To evaluate whether the increased activation of MAPK p38 and Erk1/2 induced by stimulation with L-Omp19 may be involved in the up-regulation of TNF-α, we also analyzed the effect of kinase inhibitors on the production of this cytokine. Paralleling MMP-9 results, inhibition of p38 or Erk1/2, but not Jnk1/2 (Figure 4C), significantly inhibited (*P* < 0.001) TNF-α secretion from astrocytes as elicited by HKBA and L-Omp19, and was completely abolished when both inhibitors were used in combination (Figure 6). This indicates that p38 and Erk1/2 MAPK pathways, but not Jnk1/2, could be involved in pathological responses induced by *B. abortus* and its lipoproteins in astrocytes.

### TNF-α induces MMP-9 from *B. abortus*-infected astrocytes

**Figure 7:**
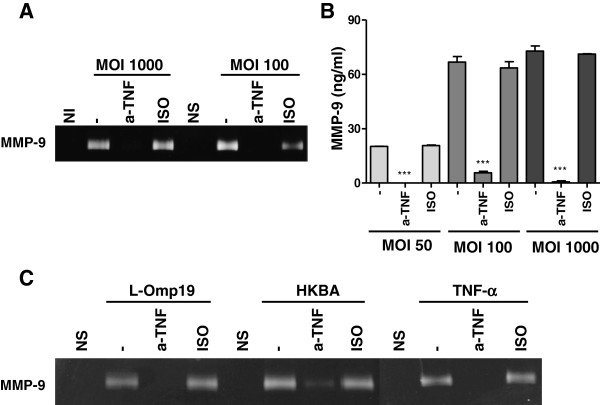
**TNF-α induces MMP-9 secretion from *B. abortus*-infected astrocytes.** Astrocytes were incubated in the presence of anti-TNF-α (a-TNF) antibody or its isotype (ISO) control before the infection of astrocytes at different multiplicities of infection (MOI) or stimulated with HKBA (10^8^ bacteria/ml), L-Omp19 (1,000 ng/ml) or TNF-α (5 ng/ml). Culture supernatants were harvested at 48 hours post stimulation to assess the expression of MMP-9 by zymography (**A**, **C**) and ELISA (**B**). Bars express the mean ± SEM of duplicates. Data shown are from a representative experiment of four performed. NI: non-infected. NS: non-stimulated. ***P < 0.001 versus untreated (minus sign).

Since a concomitant abrogation of *B. abortus*- and L-Omp19-induced TNF-α and MMP-9 production was observed when p38 and Erk1/2 pathways were inhibited and considering that TNF-α is known to induce the production of MMP-9 by other cell types infected with *B. abortus*[40], we decided to investigate the role of TNF-α in MMP-9 secretion. Astrocytes were pre-incubated with an anti-TNF-α neutralizing antibody or its isotype control and then infected with *B. abortus* or cultured with L-Omp19 or HKBA. The secretion of MMP-9 was evaluated by zymography and ELISA after culture. Recombinant TNF-α was used as control. Incubation of astrocytes with anti-TNF-α significantly inhibited (*P* < 0.001) the *B. abortus*-mediated secretion of MMP-9 at the MOI tested (Figure 7A and B). Anti-TNF-α also inhibited significantly (*P* < 0.001) the HKBA- and L-Omp19-mediated production of MMP-9 (Figure 7C). The isotype-control antibody had no effect on the response investigated. As expected, incubation of astrocytes with anti-TNF-α blocked the TNF-α-mediated MMP-9 secretion (Figure 7C). These results indicate that in astrocytes the secretion of MMP-9 mediated by *B. abortus* and its lipoproteins depends on TNF-α.

### Patients suffering neurobrucellosis display MMP-9 activity in their CSF

**Figure 8:**
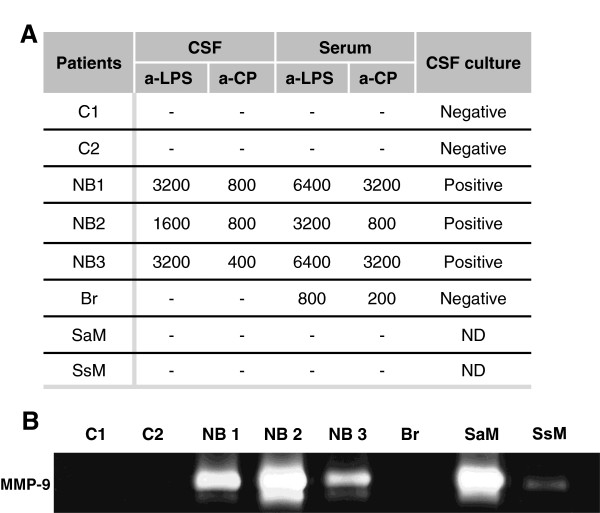
**Patients with neurobrucellosis display MMP-9 activity in their CSF.** CSF and serum samples were obtained from non-infected controls (C1 and C2), patients who had neurobrucellosis (NB1, NB2 and NB3), a patient who had brucellosis without neurological involvement (Br) and CSF samples from patients who had meningitis caused by infectious agents other than *Brucella* spp. (*Staphylococcus aureus,* SaM, and *Streptococcus* spp.*,* SsM). Antibodies against *Brucella* cytoplasmic proteins (a-CP) and LPS (a-LPS) were detected in CSF and serum samples. Culture to detect *Brucella* spp. presence was assayed in CSF (**A**). MMP-9 in CSF samples was detected by zymography (**B**). Values in Table A correspond to antibody titers against CP and LPS. ND: non-determined.

Our hypothesis was that *B. abortus* organisms that have access to the CNS can cause inflammation, and that this inflammatory response may lead to tissue damage through, at least, MMP release. To corroborate our hypothesis and give clinical relevance to our findings we investigated whether patients who developed neurobrucellosis exhibit MMP-9 activity in their CSF. MMP-9 activity, as assayed by zymography, was absent in CSF samples from non-infected controls. In contrast, MMP-9 activity was detected in the three CSF samples from neurobrucellosis patients (Figure 8B). These patients had a focalized active infection process since *Brucella* organisms were recovered from their CSF samples, which also exhibited high titers of antibodies against *B. abortus* LPS and *Brucella* cytoplasmic proteins (CP) (Figure 8A). Interestingly, no MMP-9 activity was detected in the CSF sample from a patient suffering brucellosis without neurological involvement, in which neither anti-*Brucella* antibodies nor the bacterium were detected (Figure 8A and B). This suggests that, during human brucellosis infection, MMP-9 is released to the CSF only when *Brucella* invades the CNS. Also, CSF samples from patients who had meningitis caused by infectious agents other than *Brucella* spp., exhibited MMP-9 activity (Figure 8B). These results indicate that astrocyte-secreted MMP-9 induced by *B. abortus* or its lipoproteins could be involved in the pathological manifestations of neurobrucellosis.

## Discussion

We have submitted that in neurobrucellosis, inflammation plays a key role in the mechanism of disease [6,25,41]. The importance of the inflammatory response elicited in the CNS by *Brucella* is that it can lead to irreversible CNS damage of the type that may be attributed to neuronal loss [6]. Inflammation in the CNS is thought to play a primary role in the pathogenesis of neurodegenerative diseases [42]. Given the likely contribution of inflammation to the pathogenesis of neurobrucellosis, we hypothesized that, as with some neurodegenerative diseases, these inflammatory responses may lead to CNS tissue destruction [4,5], and eventually loss of glial and neuronal cells.

Besides inflammatory cytokines, MMP play an important role in the inflammatory damage of CNS, since they can damage the brain parenchyma, the blood-brain barrier and even kill neurons [11-13]. We have already demonstrated the central role of the astrocyte in the production of pro-inflammatory cytokines that cause cell death upon infection with *B. abortus*[25]. Yet, astrocytes as a source of MMP have not been investigated in neurobrucellosis.

In this study, we present evidence indicating that upon infection with *B. abortus*, as well as other *Brucella* species, astrocytes secrete MMP-9 to culture supernatants. MMP-9 activity was evidenced by zymography and ELISA. MMP activity is counterbalanced by the action of tissue inhibitors including TIMP [38]. Upon infection or stimulation with inflammatory cytokines, TIMP production by astrocytes usually does not increase to the same extent as that of MMP-9, thus resulting in an increased MMP/TIMP ratio [43]. Supernatants from *Brucella*-infected astrocytes produced gelatin breakdown when assayed in fluid phase under native conditions in which MMP-TIMP complexes are not dissociated, as occurs during gel electrophoresis. Thus, this assay demonstrated the presence of a net gelatinase activity in these supernatants, suggesting that the MMP-9 induction detected in *Brucella*-infected astrocytes may truly contribute to collagen degradation in brain parenchyma. MMP-2, the other main metalloproteinase with gelatinase activity, has been implicated in the pathology associated with CNS infections [14]. Thus, we also investigated the up-regulation of both, MMP-2 and MMP-9 in astrocytes. However, under our experimental conditions *B. abortus* infection of astrocytes did not alter the expression of MMP-2 (not shown).

The production of MMP-9 was not dependent on bacterial viability, since it was also induced by exposure to heat-killed *B. abortus* (HKBA), suggesting that it was elicited by a structural bacterial component. We established that the structural element responsible of such response was not *B. abortu*s LPS. *B. abortus* possesses lipoproteins [44] and studies conducted in our laboratory have demonstrated that *B. abortus* lipoproteins can elicit inflammatory mediators and MMP-9 from other cell types [15,40]. Thus, we hypothesized that *B. abortus* lipoproteins could be the structural components involved in the observed phenomenon. L-Omp19, a prototypical *B. abortus* lipoprotein, induced the secretion of MMP-9 from astrocytes in a dose-dependent fashion. U-Omp19 had no effect, demonstrating that acylation of Omp19 is required for its biological activity. Not only L-Omp19 but also Pam_3_Cys was able to induce MMP-9. Since all brucellar lipoproteins likely share the Pam_3_Cys modification, this entails that any lipoprotein should be able to exert this effect. As the *B. abortus* genome contains no less than 80 genes encoding putative lipoproteins [45], many of which were shown to be expressed in the outer membrane of the bacterium [44], one can envision that the local concentration of *Brucella* lipoproteins in confined tissue spaces within the brain may be sufficient to exert their biological effects. In this context, we can hypothesize that any surface-exposed *Brucella* lipoprotein may be relevant beyond *in vitro* assays and not one lipoprotein but rather a combination of them may contribute to MMP-9 secretion elicited by *B. abortus* within the brain.

Of note, although both L-Omp-19 and *B. abortus* produce increases in MMP-9 from astrocytes, the concentration produced by L-Omp-19 is considerable higher than that produced by *B. abortus*. The reasons for this difference are unknown but may relate to the different bioavailability of L-Omp19 when used as a highly purified ligand. Lipoproteins present in whole organisms probably exert their inflammatory action either when released from living bacteria, likely in a proportion that represents only a fraction of the total amount present per organism, or when processed by phagocytic cells, as these ingest either living or dead organisms. Either way, in any such circumstance the bioavailability of *Brucella* lipoproteins would be very different when compared to when this molecule is highly pure and accessible for interaction with the innate immune receptors. This may account for the observed difference in MMP-9 between infection and L-Omp19 induction since, as we have put forward, lipoproteins are the main components of *B. abortus*-elicited inflammation [31].

Brucellar lipoproteins readily activated p38 and Erk1/2 MAPK, thus enlisting these molecules among the kinase pathways that *B. abortus* may address as it invades the CNS. Our results also demonstrate that both the p38 and the Erk1/2 MAPK pathways participate in the production of MMP-9, as elicited by HKBA and L-Omp19. The Jnk pathway, however, was not involved. Production of MMP-9 was significantly diminished either with the p38 or the Erk1/2 inhibitors, and completely abrogated when both inhibitors were used together. A concomitant inhibition of TNF-α secretion, as induced by HKBA and L-Omp19, was also achieved with the p38 or the Erk1/2 inhibitors, and again TNF-α secretion was abolished when both inhibitors were used. These results indicate that mouse astrocytes require both the Erk1/2 and p38 MAPK pathways for optimal lipoprotein-induced MMP-9 and TNF-α. A requirement for both signaling pathways for optimal TNF-α and MMP-9 responses in astrocytes has been previously reported by Ramesh *et al*. and Arai *et al*. [36,46].

Increased MMP-9 secretion is known to be induced by pro-inflammatory cytokines in a variety of CNS diseases characterized by tissue destructive pathology [24]. As TNF-α is known to be a critical factor in the pathology of neurobrucellosis [25,41], and considering that both MMP-9 and TNF-α were abrogated when MAPK signaling was inhibited in astrocytes, we investigated the role of *B. abortus*-induced TNF-α in the production of MMP-9. Blocking experiments indicated that TNF-α is sufficient for astrocyte MMP-9 secretion in response to *B. abortus* or its lipoproteins. Although IL-1β was also vindicated as an inducer of MMP-9 by astrocytes, its role, when mediated by MAPK signaling, seems to be important at physiological levels [30]. These levels were lower than the ones that we have observed when *B. abortus* infects astrocytes [25].

High CSF concentrations of cytokines and chemokines have been reported in neurobrucellosis patients [47]. However, no report has investigated the activity of MMP in the CSF of such individuals. Giving clinical relevance to our *in vitro* studies, CSF from patients suffering from neurobrucellosis exhibited MMP-9 activity as evaluated by zymography. Interestingly, MMP-9 presence in CSF seems to be a feature of an active infection process in the CNS, since a patient who had brucellosis without neurological involvement did not display MMP-9 activity in its CSF sample. This indicates that MMP-9 release is observed in CSF only when *Brucella* invades the CNS. Of note, MMP-9 activity was also present in CSF of patients suffering from meningitis due to other bacteria which are known to produce the same parenchymal inflammation of brain and spinal cord (encephalomyelitis) [48,49] as that observed in neurobrucellosis [6].

Finally, our results indicate that TNF-α stimulates MMP-9 secretion from astrocytes through the MAPK pathways. Since anti-inflammatory pyridinyl imidazole drugs such as MAPK inhibitors have been identified as putative drugs for antiinflammatory therapies [50], as have therapeutics targeting TNF-α-mediated brain inflammation [51], the data presented in this paper suggest that inhibiting such molecules (MAPK and TNF-α) may represent pharmaceutical strategies to restrict MMP-9 secretion, thereby potentially reducing morbidity associated with neurobrucellosis.

## Abbreviations

CNS: Central nervous system; MMP: Matrix metalloproteinases; MAPK: Mitogen-activated protein kinases; CSF: Cerebrospinal fluid; HKBA: Heat-killed *B. abortus*; L-Omp19: Lipidated *B. abortus* Omp19; U-Omp19: Unlipidated *B. abortus* Omp19; LPS: Lipopolysaccharide; MOI: Multiplicities of infection.

## Competing interests

The authors declare that they have no competing interests.

## Authors’ contributions

MVD and GHG conceived and designed the experiments. MCM, RS, CGS, PB, AMR, AEI, LMC, LNV and PCB performed the experiments. JC, MVD, PB and GHG contributed reagents, materials and analysis tools. MVD and GHG wrote the paper. All authors have read and approved the final version of the manuscript.
